# Cleaning patch-clamp pipettes for immediate reuse

**DOI:** 10.1038/srep35001

**Published:** 2016-10-11

**Authors:** I. Kolb, W. A. Stoy, E. B. Rousseau, O. A. Moody, A. Jenkins, C. R. Forest

**Affiliations:** 1 Wallace H. Coulter Department of Biomedical Engineering, Georgia Institute of Technology, Atlanta, GA, 30332 USA; 2 Colleges of Nanoscale Science and Engineering, SUNY Polytechnic Institute, Albany, NY, 12203 USA; 3 Neuroscience Graduate Program, GDBBS, Emory University, Atlanta, GA, 30322 USA; 4 Department of Pharmacology, Emory University School of Medicine, Atlanta, GA, 30322 USA; 5 Department of Anesthesiology, Emory University, Atlanta, GA, 30322 USA; 6 George W. Woodruff School of Mechanical Engineering, Georgia Institute of Technology, Atlanta GA, 30332 USA

## Abstract

Patch-clamp recording has enabled single-cell electrical, morphological and genetic studies at unparalleled resolution. Yet it remains a laborious and low-throughput technique, making it largely impractical for large-scale measurements such as cell type and connectivity characterization of neurons in the brain. Specifically, the technique is critically limited by the ubiquitous practice of manually replacing patch-clamp pipettes after each recording. To circumvent this limitation, we developed a simple, fast, and automated method for cleaning glass pipette electrodes that enables their reuse within one minute. By immersing pipette tips into Alconox, a commercially-available detergent, followed by rinsing, we were able to reuse pipettes 10 times with no degradation in signal fidelity, in experimental preparations ranging from human embryonic kidney cells to neurons in culture, slices, and *in vivo*. Undetectable trace amounts of Alconox remaining in the pipette after cleaning did not affect ion channel pharmacology. We demonstrate the utility of pipette cleaning by developing the first robot to perform sequential patch-clamp recordings in cell culture and *in vivo* without a human operator.

Patch-clamp recording is a gold-standard single-cell electrophysiology technique that has been widely used to discover foundational biophysical properties of excitable cells^1^. In neuroscience, the superior sensitivity and resolution of patch-clamp recording has made it an indispensable tool for discovering the tenants of ion channel activity^2^, synaptic integration^3^, plasticity^4^ and network connectivity^5^ in a variety of experimental preparations from cultured cells to living brain tissue.

There is a growing demand for large-scale measurements of brain activity on a single-cell level^6^. Patch-clamp is well-suited for such studies because it can sample electrical, morphological and genetic properties of single cells and even sub-cellular compartments^7^,^8^; however, the technique is not readily scalable. Thousands of neurons and connections must be sampled to reach conclusions about cell types and connectivity patterns in just one part of the brain^5^,^9^,^10^,^11^. While recent engineering advancements^12^ have made strides towards automating and scaling the patch-clamp technique, even to the point where a cell can be patched automatically^13^, or up to twelve cells can be patched simultaneously^14^, the highly manual nature of the technique severely limits the throughput of patch-clamp and makes large-scale studies burdensome. While planar patch-clamp systems are high-throughput, they are unsuitable for investigations of adhered cultures or living tissue^15^. Thus there is significant interest in automation and scalability of pipette-based patch-clamp recording^16^,^17^.

To achieve a successful recording, the patch-clamp pipette must have a clean tip to form a high-resistance (≥1 GΩ) junction (or gigaseal) with the cell membrane (Fig. 1a). In his Nobel Prize lecture, Edwin Neher remarked that “a gigaseal could be obtained reproducibly when suction was combined with some simple measures to provide for clean surfaces, such as using a fresh pipette for each approach and using filtered solutions”^18^. However, the need for an operator to replace the pipette for each approach is a major limitation in the following types of experiments. First, when large single- or multi-patch datasets must be collected, automation is highly desirable but cannot be achieved when pipette replacement after every trial is necessary. Second, when performing *in vivo* patch-clamp studies of sleep, audition^19^, olfaction^20^, or social interaction^21^, human operator involvement could be highly disruptive, yet unavoidable if pipettes must be replaced after every trial. Third, some promising studies are impractical at large scales because pipettes are not easily replaceable, for instance if they are coated^22^,^23^, custom-manufactured^24^,^25^, or filled with precious molecules such as synthetic peptides, novel therapeutics, human patient samples or nucleic acid constructs^26^.

We have discovered a simple, fast, and automated method for cleaning glass pipette electrodes that enables their immediate reuse. Reasoning that cleaning glass pipettes is not fundamentally different from cleaning other laboratory glassware, we found that immersing the tip into Alconox (Alconox Inc), a commercially-available detergent, at 2% w/v (20 mg/ml), for 60 seconds, permits multiple reuses in cell cultures, tissue slices, and *in vivo*. There was no detectable Alconox in the pipette after cleaning, and ion channel pharmacology results were indistinguishable between fresh and cleaned pipettes. The pipette cleaning method for the first time enables dramatically improved automation of patch-clamp, as we demonstrate with unattended, sequential patch-clamp recordings in cell culture and *in vivo*.

## Results

### Cleaning patch-clamp pipettes

**Figure 1:**
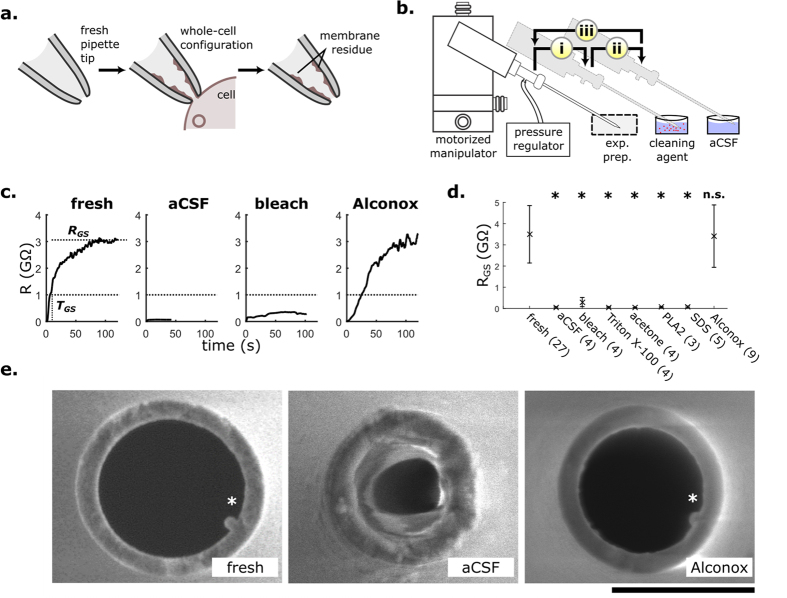
Cleaning patch-clamp pipettes (**a**). During a whole-cell patch-clamp recording, cell membrane bonds to the inner walls of the pipette. After the recording is terminated, membrane residue remains, preventing the pipette from being used for subsequent recordings. (**b**) To clean, (i) the pipette is moved from the experimental preparation to a wash bath where a cleaning agent is cycled within the tip, (ii) then to a rinse bath where the remaining cleaning agent is expelled into aCSF, (iii) and returned back to the experimental preparation. (**c**) Representative gigaseal formation traces. When using contaminated pipettes cleaned with Alconox, a multi-GΩ seal forms reliably, as would be expected when using a fresh pipette. On the other hand, cleaning with aCSF (artificial cerebrospinal fluid) and bleach does not result in gigaseal formation. R_GS_: maximum gigaseal resistance, T_GS_: time (s) to reach 1 GΩ (horizontal dashed line) (**d**). Of the six tested detergents and aCSF, only Alconox reliably achieved gigaseal resistances comparable to those of fresh pipettes (p < 0.001; one-way ANOVA with Dunnett’s post-hoc test. *p < 0.001; n.s.: not significant, p > 0.9). Data shown as mean ± s.d.; n for each cleaning agent is shown in parentheses. PLA2: Phospholipase A2; SDS: Sodium Dodecyl Sulfate. (**e**) Scanning Electron Microscopy (SEM) images of pipette tips. The Alconox-cleaned pipette tip resembles that of the fresh pipette. On the other hand, the pipette tip cleaned with aCSF is visibly contaminated with cell membrane residue. Pipette filament denoted with *. Scale bar: 1 μm.

After a recording attempt in an experimental preparation, pipette cleaning is accomplished in three steps. First (Fig. 1b,i), the pipette is moved to a bath containing a cleaning agent. We reasoned that a cleaning agent could be pneumatically taken up into the pipette tip in order to remove adherent cellular debris resulting from a previous patch-clamp trial. The height of a typical lipid ‘bleb’ that forms in the pipette tip during a gigaseal is 30–60 μm^28^. When applying a high vacuum (−300 mBar) to the pipette, fluid is taken up at a rate of ~760 pL/s based on pipette inner diameter of 1 μm^27^. To reach a height of 60 μm in the pipette, 6.2 pL of fluid must be taken up, which would theoretically require approximately 8.2 ms; however, to account for tubing compliance and to ensure the cleaning agent reaches the bleb height at a high concentration, we initially apply the vacuum for 4 s, which draws up 3 nL of the cleaning agent. After this initial uptake, the cleaning agent is pneumatically cycled within the pipette tip (−300 mBar, +1,000 mBar for 1 s each, 5 repetitions total) to physically agitate glass-adhered tissue. To expel the original cleaning agent volume (3 nL) out of the pipette at 1,000 mBar, only 1.3 s are theoretically required. We add a ~10x safety factor to ensure the removal of cleaning agent molecules that may have diffused further up the pipette. We thus expel the fluid for 10 s. In the second step of pipette cleaning (Fig. 1b, ii), the pipette is moved from the cleaning bath to a bath containing standard artificial cerebro-spinal fluid (aCSF). Any remaining cleaning agent in the pipette is expelled (+1,000 mBar for 10 s). Lastly (Fig. 1b,iii), the pipette is moved back to the experimental preparation. Pipettes can then be immediately reused for a subsequent patch-clamp attempt. Refilling a pipette with internal solution is unnecessary because pipettes are commonly filled with enough solution for hundreds of attempts. All moving and cleaning steps together require 60 seconds.

Alconox was the only cleaning agent that enabled pipettes to be reused. We performed whole-cell patch-clamp recordings on human embryonic kidney (HEK293T) cells using fresh (previously unused) pipettes, cleaned them with one of six different commonly available glassware cleaning agents (Supplementary Table 1) as well as a control solution (aCSF), and attempted to patch another cell with the same pipette. On the second attempt, pipettes cleaned with Alconox, but not with any other cleaning agent, produced gigaseal resistances (R_GS_) that were not significantly different from those produced by fresh pipettes (Fig. 1c,d). Successful whole-cell recording rates were not different between fresh and Alconox-cleaned pipettes (fresh = 92.6%, Alconox-cleaned = 88.9%, p = 0.87, Fisher’s exact test; other detergents and aCSF = 0%). Gigaseals were held for 1–2 min to ensure stability. No gigaseals were spontaneously lost during this time in either fresh or Alconox-cleaned pipettes. When using Alconox-cleaned pipettes, an outside-out patch reliably formed after withdrawing the pipette from the cell, as would be expected if using fresh pipettes^1^. Further, pre-cleaning fresh pipettes in Alconox had no effect on gigaseal formation (Supplementary Fig. 1). Using scanning electron microscopy (SEM), we confirmed that the tip of the pipettes was being cleaned using Alconox but not aCSF (Fig. 1e). Unless stated otherwise, all subsequent experiments used Alconox.

**Figure 2:**
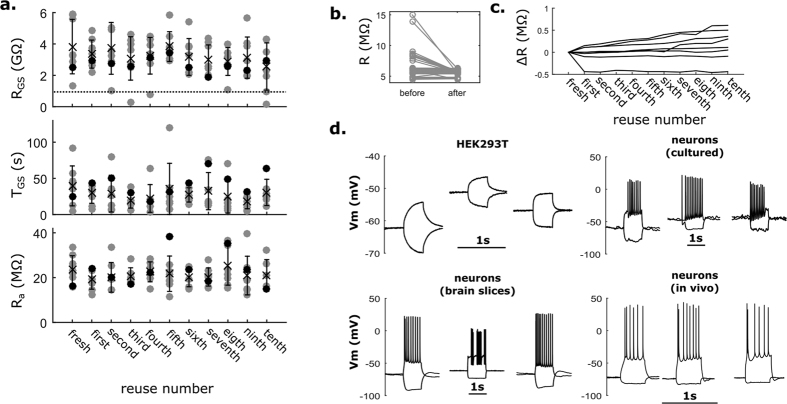
Pipettes can be successfully reused ten times. (**a**) Recording quality parameters R_GS_, T_GS_ (defined in Fig. 1c) and R_a_ (whole-cell access resistance) do not decrease over ten reuses of the same pipette (n = 8 pipettes). Dashed line: 1 GΩ (threshold for gigaseal). Gray circles: individual trials; black circles: representative experiment with a single pipette. Data shown as mean ± s.d. (**b**) Pipette resistance before and after cleaning with Alconox (n = 88 pairs, 8 pipettes). (**c**) Change in pipette resistance from first to tenth reuse. After 10 reuses, resistance changed (ΔR) by median: 0.175 MΩ, max: 0.61 MΩ, min: −0.44 MΩ from fresh pipette. (**d**) Representative whole-cell responses to step current injections in different experimental preparations. Recordings from HEK293T cells were obtained from the representative experiment (black circles) in (**a**). In each set, a single pipette is used for all three whole-cell recordings. In all preparations pipettes were reused up to ten times.

Pipettes could be reused 10 times consecutively with no degradation in recording quality if they were cleaned between each patch-clamp attempt (Fig. 2a). We successfully patched 84 HEK293T cells (of n = 88 attempts including 1 fresh and 10 reuses; success rate = 95%) using 8 pipettes, and found no effect of the number of reuses on gigaseal resistance (R_GS_), the time to reach 1 GΩ (T_GS_), and the access resistance of the patched cell (R_a_) for each trial (one-way repeated-measures ANOVA with number of reuses (1–10) as predictor; R_GS_: F_10,40_ = 0.99, p = 0.46, n = 8; T_GS_: F_10,40_ = 0.36, p = 0.96, n = 5, R_a_: F_10,40_ = 0.72, p = 0.70, n = 5). Notably, even when pipettes failed to reach a gigaseal (e.g., R_GS_ < 1 GΩ on third, fourth reuse in Fig. 2a), they were successfully cleaned and reused again, suggesting that gigaseal failures did not irreversibly contaminate the pipette tip. Beyond 10 reuses, we did not perform a systematic study of pipette longevity; however, a pilot experiment confirmed our suspicion that pipettes could not be reused indefinitely, when three consecutive failed patch-clamp attempts occurred after 26 reuses (Supplementary Fig. 2).

Electrical resistance is commonly used to estimate pipette tip opening size prior to performing the patch-clamp recording. Higher resistances indicate a smaller opening. Therefore, increases in electrical resistance could be caused by obstructions on the pipette tip. To assess whether cleaning reduced the amount of obstructions, we measured resistance twice: once immediately after completing a whole-cell recording (before cleaning) and once more after cleaning. As expected, pipette resistance before cleaning was higher than after (Fig. 2b; before: median = 5.93 MΩ, range: 4.49 to 15.0 MΩ; after: median = 5.77 MΩ, range = 4.46 to 6.28 MΩ; p < 0.05, Wilcoxon signed-rank test). Over 10 reuses, the resistance of the pipette did not change significantly (Fig. 2c; p ≥ 0.19 for 1^st^–10^th^ reuse, n = 8, Wilcoxon signed-rank test). Together, these results suggest that cleaning eliminates residue obstructions on the pipette tip over ten reuses.

Using a single pipette for each experimental preparation, we were able to repeatedly patch-clamp in neuron cultures, acute brain slices, and *in vivo* (Fig. 2d). Cleaning did not visibly alter action potential generation in these experiments. In brain slices, successful gigaseals and successful whole-cell recordings were attained at similar rates between fresh and cleaned pipettes (gigaseals: fresh = 14/18 (78%), cleaned = 38/46 (82%), p = 0.73; whole-cell recordings: fresh = 7/18 (39%), cleaned = 22/46 (48%), p = 0.58, Fisher’s exact test). We recorded from neurons for 20 min, and found that the access resistance, input resistance, and holding current remained stable in most cells across that time (Supplementary Figs 3–5), indicating that whole-cell recordings obtained using cleaned pipettes are stable over the duration of a typical experiment.

### Verifying detergent removal after pipette cleaning

A major concern of using Alconox to clean pipettes is that residual surfactants in the pipette could damage the cell or affect its normal biophysical activity during a patch-clamp recording. Alconox is composed of 33–43% sodium bicarbonate, 10–20% sodium (C10-C16) linear alkylbenzene sulfonate (LAS), 5–15% sodium tripolyphosphate, 5–15% tetrasodium pyrophosphate, 1–10% sodium carbonate, and 1–5% sodium alcohol sulfate^29^. We chose LAS, a commonly-used surfactant, as a proxy for measuring Alconox concentration remaining after rinsing.

**Figure 3:**
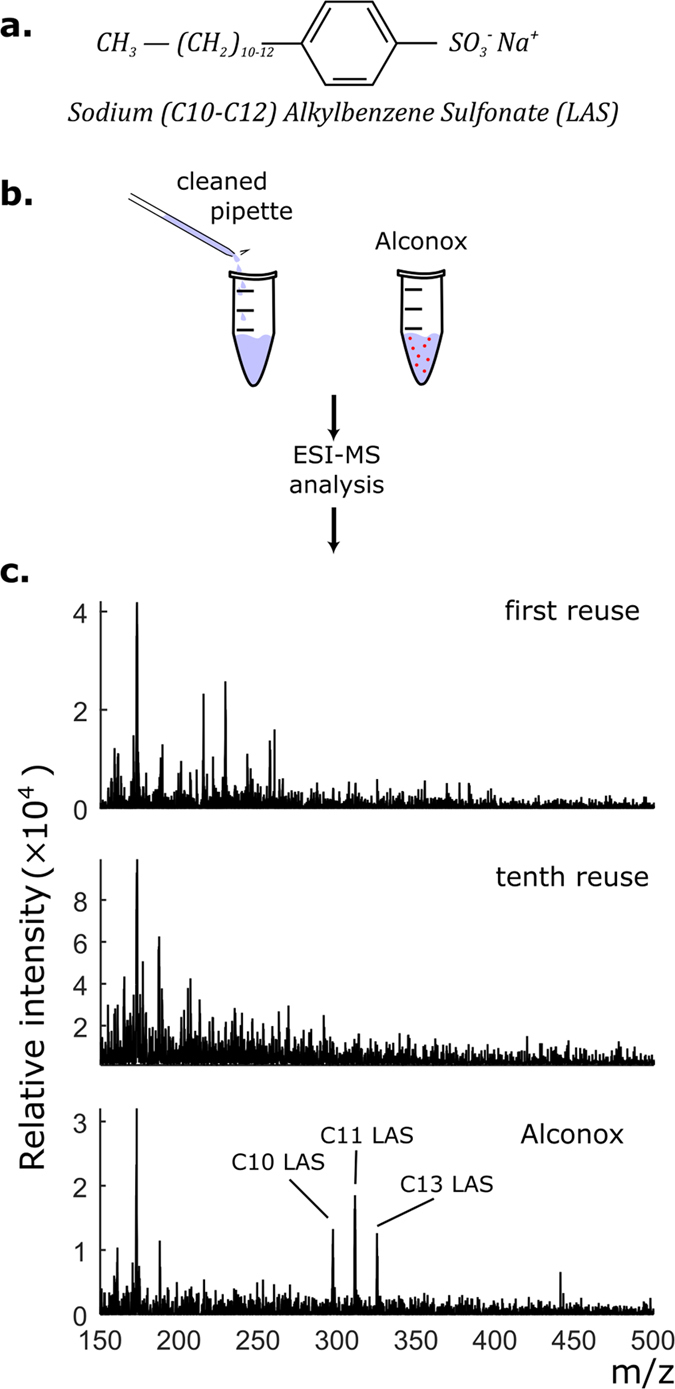
Detection of linear alkylbenzene sulfonate (LAS) in cleaned pipettes using electrospray ionization mass spectrometry (ESI-MS). (**a**) LAS is the prevalent cytotoxic ingredient in Alconox. Alconox is composed of LAS with carbon chains lengths 10–12 (C10-C12). (**b**) The contents of cleaned pipettes were collected in vials after either one or ten reuses. Known amounts of Alconox were analyzed to find the detection limit. (**c**) ESI-MS spectrum. C10-C12 LAS is not detectable in pipettes reused once or ten times (top, middle) but is detected in an Alconox solution (174 ng/mL Alconox in DI water, bottom), indicating that less than 174 ng/mL of Alconox remains in the pipettes after cleaning. C10: expected m/z: 297.1, found: 297.3; C11: expected m/z: 311.2, found: 311.2; C12: expected m/z: 325.2, found: 325.1.

We measured the amount of Alconox remaining after cleaning using electrospray ionization mass spectrometry (ESI-MS). Only C10-C12 LAS was found in significant quantities in the Alconox solution (Supplementary Fig. 6), so we focused our analysis on this family of compounds (Fig. 3a). We found no traces of LAS in pipettes cleaned once or ten times (Fig. 3b,c (top, middle)), while the instrumentation detection limit was found to be 174 ng/mL (Fig. 3c (bottom)). Thus, less than 174 ng/mL of Alconox remained in the pipettes after the cleaning procedure. As a control, we ensured that LAS could still be detected using ESI-MS when purposefully introduced in small concentrations into the pipette (Supplementary Fig. 6).

**Figure 4:**
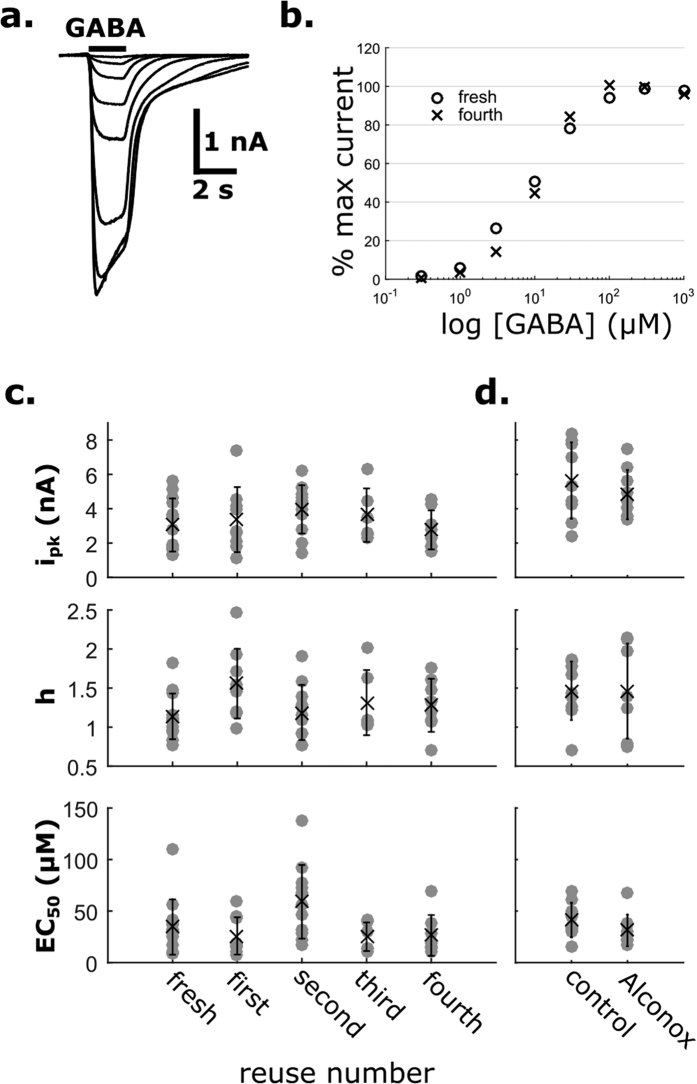
Pipette cleaning in conjunction with GABAAR pharmacology. (**a**) Representative current traces recorded by whole-cell patch-clamp recording of HEK293T cells transfected with α1β2γ2s GABA_A_Rs. Black bar denotes GABA application (**b**) Representative normalized peak current responses to different GABA concentrations. The response captured with a fresh pipette is similar to that captured with a used pipette (fourth reuse). (**c**) Dose response characteristics of cells patched with fresh and reused pipettes. i_pk_: peak evoked current; h: Hill coefficient; EC_50_: half-maximal response concentration. No change in the three characteristics is observed over four reuses. (**d**) A low dose of Alconox (67 μg/mL, or equivalently, 385× the detection limit of remaining Alconox in the pipette after cleaning) in the internal solution does not affect dose response characteristics.

Any effect of trace amounts of Alconox in cleaned pipettes on cell receptor pharmacology would be highly undesirable. Residual LAS could disrupt gigaseal formation, thus decreasing signal quality or, more subtly, interact with amphipathic allosteric modulatory pockets on receptors, thus covertly compromising pharmacological experiments. The γ-aminobutyric acid type A Receptor (GABA_A_R) is highly sensitive to *extracellular* application of surfactants^30^, including LAS^31^; however, its *intracellular* effects have not been thoroughly studied. Nevertheless, we reasoned that GABA_A_R could serve as an indicator of adverse effects of Alconox. We used HEK293T cells expressing GABA_A_R as a model system to verify that cleaned pipettes are pharmacologically inert. We whole-cell patch-clamped using fresh and cleaned pipettes, and fit the measured whole-cell current responses to increasing concentrations of GABA^32^ (Fig. 4a) to the Hill equation. Neither the peak evoked current (I_pk_), the Hill’s coefficient (h), nor the half maximal effective concentration (EC_50_) metrics changed as a function of reuses (linear regression model, H_0_: slope ≠ 0; I_pk_: p = 0.998, 95% CI: −318 to 317 pA; h: p = 0.719, 95% CI: −0.065 to 0.093; EC_50_: p = 0.751, 95% CI: −6.75 to 4.90). This finding demonstrates that GABA concentration-response curves of the patch-clamped cells do not change on a population level as a function of the number of times a pipette has been reused (Fig. 4b,c). Overall, pharmacology results obtained using fresh pipettes were indistinguishable from those obtained using cleaned pipettes, suggesting no effect of trace amounts of Alconox on normal receptor function of GABA_A_Rs.

We also found that even if small amounts of Alconox remain in the pipette after cleaning, they do not impact gigaseal formation or GABA_A_R pharmacology. When 67 μg/mL of Alconox (385× the ESI-MS detection limit) was intentionally dissolved in the pipette internal solution, gigaseals were still reliably achieved in all tested pipettes (n = 9). No pharmacological difference between these pipettes and fresh ones was detected (Fig. 4d; I_pk_: p = 0.37; h: p = 0.99; EC_50_: p = 0.19). On the other hand, when a much larger dose, 10 mg/mL Alconox (0.5× the concentration in the wash bath) was added to the pipette, gigaseals did not form and the targeted cell exhibited clear apoptotic blebbing (Supplementary Fig. 7).

### Unattended, sequential patch-clamp recording

Previous efforts have partially or fully automated the process of obtaining a single whole-cell recording *in vitro*^14^,^33^,^34^ as well as *in vivo*^16^,^35^; however, in all of these studies, a trained operator was needed to replace pipettes to start another attempt. Removing this manual step would enable unattended patch-clamp experiments and improve scalability and throughput. To explore this, we integrated the pipette cleaning algorithm into our previously-developed Autopatcher software. We tested the resulting robot, which we call ‘patcherBot’ by first performing patch-clamp recordings in HEK293T cells. After the operator selected candidate cells for patch-clamp recording, the patcherBot obtained successful gigaseals in 9/10 attempts, and successful whole-cell recordings in 6/10 attempts over the span of 33 minutes of unattended operation using a single pipette (Supplementary Video 1). We also deployed the patcherBot to perform blind, *in vivo* patch-clamp recordings in the mouse barrel cortex (Supplementary Video 2). Using four pipettes, the robot obtained successful gigaseals in 13/34 attempts, and successful whole-cell recordings in 10/34 attempts over a total span of 171 minutes *in vivo* (Fig. 2d).

## Discussion

Discovering that pipettes could be reliably cleaned and reused multiple times was surprising given the dogmatic, decades-long practice of replacing pipettes after each patch-clamp attempt. Our simple, fast, and automated procedure consists of dipping pipettes into a commercially-available detergent, Alconox, followed by rinsing in aCSF. We demonstrated the effectiveness of this cleaning method by reusing pipettes 10 times, with no decrease in whole-cell recording quality in cultured cells, acute brain slices, and *in vivo*. After cleaning, the residual Alconox mass concentration in the pipettes was quantified to be less than 174 ng/mL, and shown to have no pharmacological effect on GABA_A_Rs. Since pipette cleaning is automatic, we integrated it into the Autopatcher to perform unattended, sequential patch-clamp recordings on HEK293T cells *in vitro* and neurons *in vivo*.

Alconox is composed of a surfactant, emulsifier and water softener, and in solution, it interacts with cell membranes bound to the glass pipette tip. These three ingredients solubilize adhered lipids, stabilize the lipid micelles in solution, and enhance surfactant effectiveness, respectively. After testing various cleaning agents, we chose Alconox based on empirical observation; it was not evident from chemical principles why only Alconox sufficiently cleaned pipettes to enable their reuse. Interestingly, Kao *et al*. used bleach to successfully clean planar borosilicate patch-clamp chips up to five times for whole-cell recording^36^; yet in our experiments using conventional pipettes, bleach did not work, suggesting that the geometry of the experimental preparation may influence cleaning effectiveness. Overall, the precise mechanism of how cell membranes bond with glass during a gigaseal is still not understood despite notable efforts^28^,^37^, making it difficult to devise a cleaning strategy from chemical principles. Indeed, our cleaning procedure, while functional, was not thoroughly optimized to minimize cleaning time and maximize the number of reuses. A detailed biochemical study of gigaseal formation could greatly inform such optimization efforts.

While we have demonstrated that cleaning does not affect the ion channel pharmacology of GABA_A_Rs, we have not exhaustively tested the method in cells expressing other proteins. While several studies have characterized the effects of extracellularly applied detergents on various receptors^31^,^38^,^39^, the intracellular effect of LAS in small concentrations is not well-understood. Thus to verify general applicability, the method should be validated using more channels, receptors, and cell types.

For many patch-clamp experiments, pipette internal solution contains ingredients that degrade over time if not refrigerated (e.g. Adenosine 5′-triphosphate magnesium salt, Guanosine 5′-triphosphate sodium salt hydrate, phosphocreatine); thus, we would expect that pipettes after multiple cleanings will have reduced concentrations of these ingredients. We hypothesize that maintaining a chilled (i.e., 4 °C) environment for pipette and internal solution, or devising a method for replacing internal solution after every trial could mitigate this deleterious effect.

The cleaning process is typically faster than manually swapping pipettes (i.e., 1 min versus approximately 2 min) and does not depend on operator experience, dexterity, or fatigue level. It requires no complex hardware additions to existing electrophysiology setups and no expensive or caustic reagents. Therefore, it can be readily integrated into different experimental preparations and coupled with existing techniques that complement patch-clamp such as extracellular stimulation^40^, two-photon microscopy^41^, and optogenetics^42^. Pipette cleaning also facilitates experiments requiring specialized pipette tips. Various tip polishing techniques (using heat, pressure^24^,^43^, or focused ion beam^25^) and coatings^23^,^44^ can improve the ability to obtain recordings and their quality; however, these techniques are typically too time-consuming to be routine. The ability to reuse these custom-shaped pipettes could make these involved techniques more practical and scalable, reducing the need for automated pipette fabrication^45^ and inspection^46^. Cleaning could also greatly facilitate simultaneous multi-patch (i.e. dual, quadruple, octuple, etc.) experiments that have been instrumental in elucidating single-cell connectivity patterns in the brain. Since cleaning is faster than manual pipette replacement and requires no human intervention, it could increase the number and duration of simultaneous recordings.

In creating the patcherBot, we demonstrate the first robot to successfully patch-clamp multiple cells sequentially without human intervention, opening the door for walk-away automated experiments. In addition to automation, the patcherBot could be deployed in experiments where human involvement is deleterious. For example, it could be utilized *in vivo* in sensitive behavior experiments to minimize potential noise or odorant confounds that could be introduced by the experimenter.

To encourage the dissemination of this technology, we provide all hardware schematics, including cleaning dish blueprints, pressure control box design, and associated cleaning software free online at www.autopatcher.org/cleaning.

## Methods

### Pipette cleaning method

We made minor modifications to standard *in vitro* and *in vivo* electrophysiology rigs to enable them to be used for pipette cleaning. We manufactured an electrophysiology recording chamber with two wells for the cleaning and rinse solutions. A variant of this chamber without the main recording well was manufactured for use *in vivo*. We programmed a custom-built and a commercially-available pressure control system (Autopatch 1500, Neuromatic Devices) to deliver timed pressure pulses to the pipette during the cleaning and rinsing steps. For pipette cleaning, we chose the maximum intake and expel pressures (−300 mBar, +1,000 mBar) that could be produced by our regulators (intake: 990-005203-005, Parker; expel: 990-005101-015, Parker). Different pressure regulators can also be used. We also programmed standard 3-axis micromanipulators to move the pipette automatically between the experimental preparation location and the two baths. Finally, we created a graphical user interface (GUI) in LabVIEW (National Instruments) that starts the automatic cleaning procedure with a button click. For the patcherBot robot, the pipette cleaning module was integrated into the Autopatcher software algorithm^13^.

### HEK293T cell preparation

Human embryonic kidney (HEK293T) cells (American Type Culture Collection, Manassas, VA) were maintained at 37 °C and 5% CO2 in Eagle Minimum Essential Medium (MEM) supplemented with 5% FBS, 40 μM L-glutamine, 100 U/ml penicillin and 0.1 mM streptomycin. Cells were passaged regularly and split when they reached 70% confluency. Cells used for *in vitro* electrophysiology experiments were grown on glass coverslips (12 × 12 mm, No. 2, VWR). For the pharmacology experiments, HEK293T cells were transfected with α1β2γ2 GABAA receptors and GFP using X-tremeGENE (Roche Diagnostics). Patch-clamp experiments were performed 24–72 hours post-transfection in aCSF consisting of (in mM): 161 NaCl, 10 HEPES, 6 D-Glucose, 3 KCl, 1 MgCl_2_, 1.5 CaCl_2_ (pH: 7.4). The internal pipette solution consisted of (in mM): 120 KCl, 2 MgCl_2_, 10 EGTA, 10 HEPES (pH: 7.2–7.3, osmolarity: 290–300 mOsm).

### Primary neuron culture

Primary cortical neuronal cultures from E18 Sprague Dawley rat embryos were purchased from Brainbits, LLC preplated on glass coverslips at 16000 cells/cm^2^. Cortical tissues were triturated with a Pasteur pipette, and then were plated onto 12-mm glass coverslips coated with poly-D-lysine. Culture media (NbActiv4, Brainbits, LLC) consisted of Neurobasal/B27 supplemented with creatine, estrogen, and cholesterol. Neurons were maintained at 37 °C in a humidified incubator gassed with 95% air and 5% CO_2_. Patch-clamp experiments were performed at 8–12 days *in vitro* (DIVs) in aCSF consisting of (in mM): 126 NaCl, 3 KCl, 2 CaCl_2_, 1.5 MgSO_4_, 1 NaH_2_PO_4_, 25 NaHCO_3_, 25 D-glucose, saturated with 95% O2 and 5% CO2 (pH 7.4). Internal solution consisted of (in mM): 100 K-gluconate, 30 KCl, 10 HEPES, 2 MgSO_4_, 0.5 EGTA and 3 Mg-ATP (pH 7.4).

### Brain slice preparation

All animal procedures were in accordance with the US National Institutes of Health Guide for the Care and Use of Laboratory Animals and were approved by the Institutional Animal Care and Use Committee at the Georgia Institute of Technology. Acute brain slices were prepared from male C57/BL6 mice (aged P30-P60) using the protective recovery method described in detail elsewhere^47^. Animals were heavily anesthetized with isofluorane and perfused trans-cardially with NMDG solution containing (in mM): 93 N-methyl-D-glucamine (NMDG), 2.5 KCl, 1.2 NaH_2_PO_4_, 30 NaHCO_3_, 20 HEPES, 25 Glucose, 5 Na-ascorbate, 3 Na-pyruvate, 10 MgSO_4_.7H_2_O, 0.5 CaCl_2_.2H_2_O (pH: 7.3–7.4, osmolarity: 300–310 mOsm). Mice were quickly decapitated, the brain was extracted, embedded in 2% agarose and cut into 300 μm coronal slices. The slices were incubated at 34 °C in the cutting solution for 10–12 minutes and subsequently transferred to a recovery solution containing (in mM) 92 NaCl, 2.5 KCl, 1.2 NaH_2_PO_4_, 30 NaHCO_3_, 20 HEPES, 25 Glucose, 5 Na-ascorbate, 2 Thiourea, 3 Na-pyruvate, 2 MgSO_4_.7H_2_O, 2 CaCl_2_.2H_2_O (pH: 7.3–7.4, osmolarity: 300–310 mOsm) for at least 60 minutes prior to recording. The recording aCSF consisted of (in mM): 124 NaCl, 2.5 KCl, 1.2 NaH_2_PO_4_, 24 NaHCO_3_, 5 HEPES, 12.5 Glucose, 2 MgSO_4_.7H_2_O, 2 CaCl.2H_2_O. The internal pipette solution consisted of (in mM): 135 K-Gluconate, 10 HEPES, 4 KCl, 1 EGTA, 0.3 Na-GTP, 4 Mg-ATP, 10 Na_2_-phosphocreatine (pH: 7.2–7.3, osmolarity: 290–300 mOsm).

### *In vitro* patch-clamp recording

Recordings in HEK cells, cultured neurons and brain slices were performed using standard *in vitro* electrophysiology systems (either SliceScope Pro 1000, Scientifica Ltd. or Axiovert 200, Zeiss). Borosilicate pipettes were pulled on a horizontal puller (P-97, Sutter Instruments) to a resistance 4–8 MΩ. Signals were acquired using Multiclamp 700B (Molecular Devices), and digitized at 10 kHz using a data acquisition board (NI USB-6221, National Instruments or Digidata 1322A, Molecular Devices). The data were saved to a computer using either a custom program written in LabVIEW or pClamp 9 (Molecular Devices).

For pharmacology experiments, drugs were applied using a rapid solution changer (RSC-160, BioLogics Science Instruments) connected to a 10-channel infusion pump (KD Scientific Inc) and controlled by pClamp 9. GABA concentration-response assays (DRCs) were performed by exposing each whole-cell patch to 8 increasing concentrations of GABA (0.3, 1, 3, 10, 30, 100, 300 and 1,000 μM) for 2 s with an 8 s washout between concentrations. Whole-cell currents were recorded in voltage clamp mode at −60 mV, digitized at 200 Hz and filtered at 100 Hz. Peak current responses to the concentrations were fit to the Hill equation:


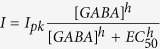


where *I* is the peak current, *I*_*pk*_ is the maximal evoked current, *EC*_50_ is the half-maximal response concentration, and *h* is the Hill coefficient. Adding Alconox to the internal solution (Fig. 4d) did not appreciably change the pH or the osmolarity of the solution (∆pH = 0.02, ∆Osm = 8 mOsm).

For cell quality measurements (Fig. 1), seal resistance was recorded at 10 Hz from the moment of positive pressure release on a target cell, and until the gigaseal (defined as R ≥ 1GΩ) was held for at least 1 min. Seal resistance traces were smoothed with a 2.5 s moving average filter. The resistance at the end of the 1 min time interval was considered to be the ultimate gigaseal resistance (R_GS_). The time to reach the gigaseal (T_GS_) was calculated as the time elapsed between R = 20 MΩ and R = 1 GΩ if the gigaseal was successful; otherwise, T_GS_ was undefined and not included in analysis. In HEK cells and brain slices, access resistance (R_a_) was measured in voltage clamp mode after pipette capacitance compensation, using a custom-written LabVIEW algorithm. In brain slices, a whole-cell was considered successful when the holding current was ≥ −250 pA, and access resistance was stable.

### *In vivo* Autopatching

Methodology to perform automated whole cell patch-clamp electrophysiology has been previously described^48^. Briefly, 3 male C57/BL6 mice (aged P30-P60) were anesthetized and maintained using isoflurane with a vaporizer (Datex-Ohmeda Isotec 5) at 1% concentration and checked periodically for response to toe-pinch. Mice were affixed in a stereotaxic apparatus (Kopf) and a head plate implant attached, followed by opening of a craniotomy 1 mm wide above the barrel cortex (centered 0.83 mm posterior and 3.0 mm lateral to bregma) using previously described protocols^49^. Once opened, the craniotomy was superfused with sterile saline throughout the experiment to keep the tissue moist. The anesthetized animals were then head-fixed using a custom holder composed of optical posts and plate clamps (Eksma Optics 830-0055). Pipettes, internal solution, amplification and digitization were the same as in the *in vitro* preparation.

### Scanning Electron Microscopy

To prepare pipettes for SEM imaging, the internal solution was first drained using a micropipette (Eppendorf epTIPS, 20 μL). Pipettes were then tip-filled with DI water for 60 s by applying a strong vacuum (−300 mBar) in order to dilute any remaining internal solution at the tip and prevent the formation of salt crystals that would compromise image quality. Pipettes were then placed tip-down in a dessicator and left to dry overnight. They were then mounted to a custom-made sample holder and imaged with a Hitachi SU8230 SEM.

### ESI-MS

Preparation of samples for ESI-MS was performed no more than 2 days prior to analysis. Standard patch pipettes were filled with 20 μL de-ionized (DI) water and cleaned using the standard cleaning procedure either once or ten times consecutively. In order to minimize potential noise in the intensity signal due to cellular debris, these pipettes were not used for patch-clamp recordings. For Supplementary Fig. 4, 67 μg/mL Alconox was added to the pipette internal solution. Pipette tips were carefully broken and all fluid was collected into microcentrifuge tubes (Safe-Lock, Eppendorf) under positive pressure (200 mBar). To find the detection limit, we created concentration standards by dissolving Alconox in DI water at 17.4, 174, 1,174 and 11,740 ng/mL. The pipette fluid and the Alconox standards were then transferred to individual vials (Waters Technologies) for analysis.

ESI-MS analysis was performed using Micromass Quattro LC in negative-ion mode. For each vial, the sample was infused at a rate of 10 μL/min and mixed with 50:50 water:acetonitrile with 10 mM ammonium acetate. The cone and capillary voltages were varied to get the best intensity for the [M-H]- ion, and then the collision voltage was varied to get the highest intensity for the product ion.

## Additional Information

**How to cite this article**: Kolb, I. *et al*. Cleaning patch-clamp pipettes for immediate reuse. *Sci. Rep.* **6**, 35001; doi: 10.1038/srep35001 (2016).

## Supplementary Material

Supplementary Video 1

Supplementary Video 2

Supplementary Information
