# Supplementary material for: Cleaning patch-clamp pipettes for immediate reuse
Source: Sci Rep. 2016 Oct 11;6:35001. doi: 10.1038/srep35001 (PMC5057089; doi:10.1038/srep35001)
Supplement: Supplementary Information [file srep35001-s3.docx]

**Cleaning patch-clamp pipettes for immediate reuse**

I Kolb, WA Stoy, EB Rousseau, OA Moody, A Jenkins, CR Forest

**Supplementary Information**

**SUPPLEMENTARY INFORMATION**

**
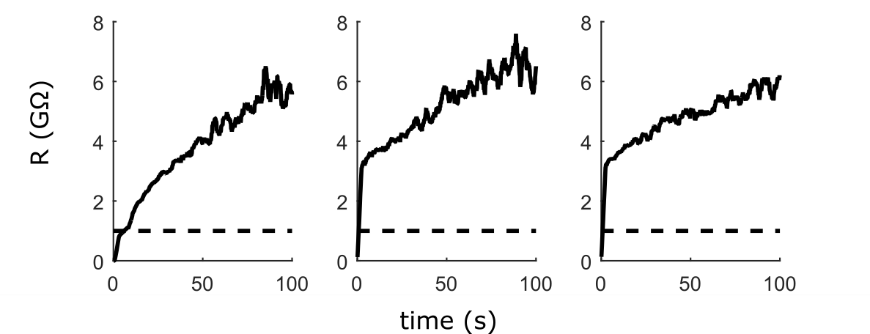
**

**SUPPLEMENTARY FIGURE 1:** Three representative gigaseals obtained with fresh pipettes that were pre-cleaned in Alconox. Pre-cleaning did not hinder the formation of stable gigaseals.


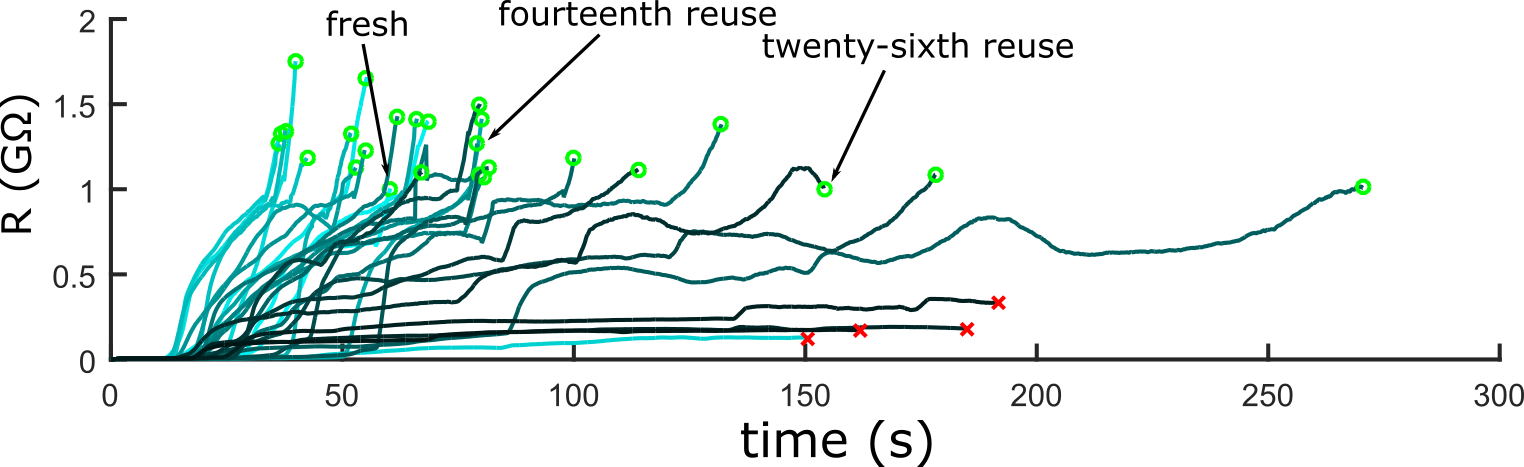


**SUPPLEMENTARY FIGURE 2**: Gigaseal resistances of thirty reuse attempts with a single pipette. The pipette was successfully reused twenty-six times (green circles: R > 1 GΩ). The pipette did not reach a gigaseal on the fifth reuse, and consecutively on twenty-seventh through thirtieth reuse (red circles: R < 1 GΩ). Gigaseal attempts are color-coded from light to dark according to the number of times the pipette was used. After approximately fourteen reuses, the time to form a gigaseal increases.


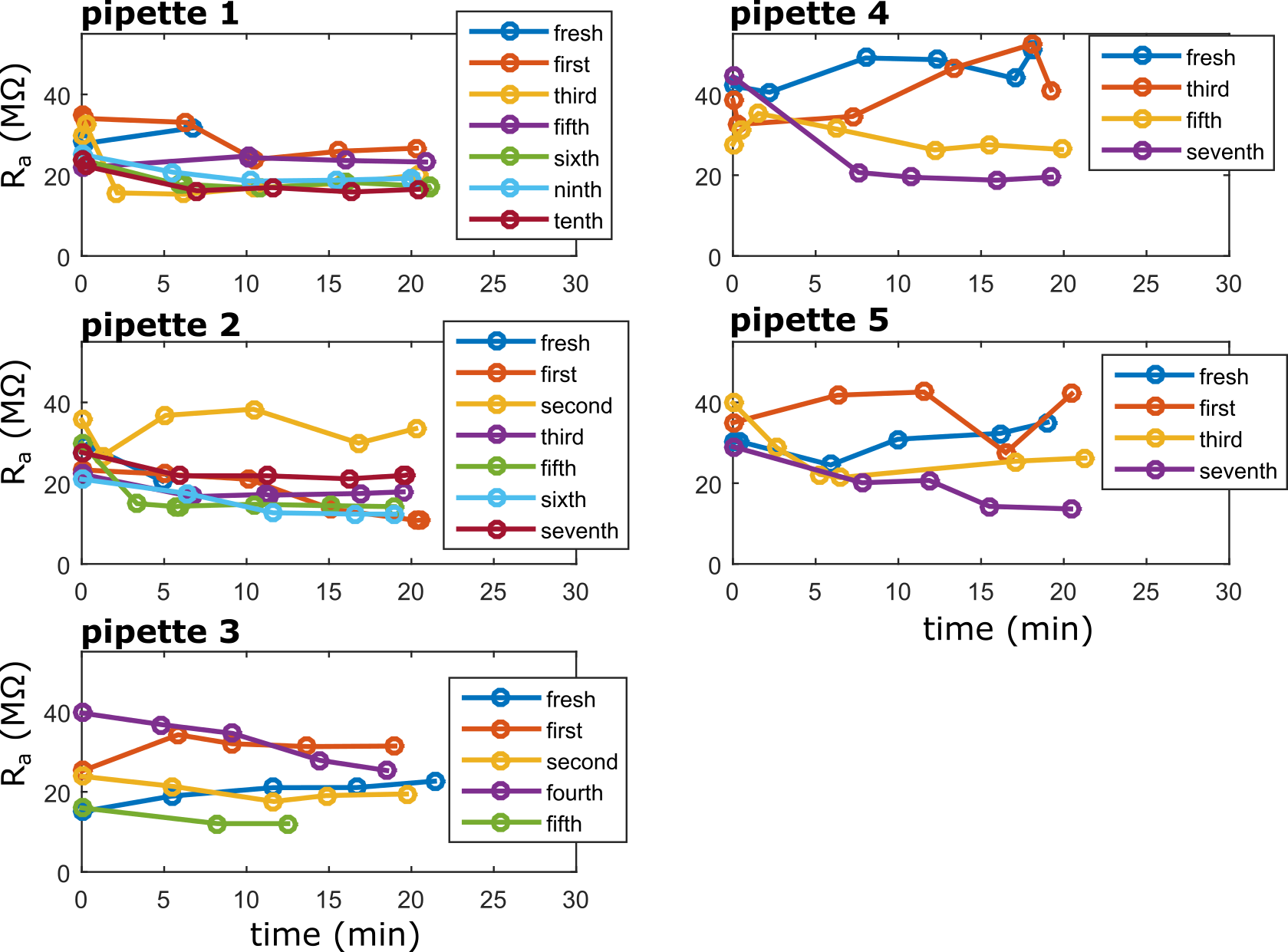


**SUPPLEMENTARY FIGURE 3**: Access resistance of neurons in brain slices. For each plot, a single pipette was used. Only successful attempts (resulting in whole-cell recordings) are shown. Cells were held for ~20 minutes. Access resistance did not increase for fresh pipettes as well as for pipettes reused 1-10 times, indicating stable whole-cell recordings.


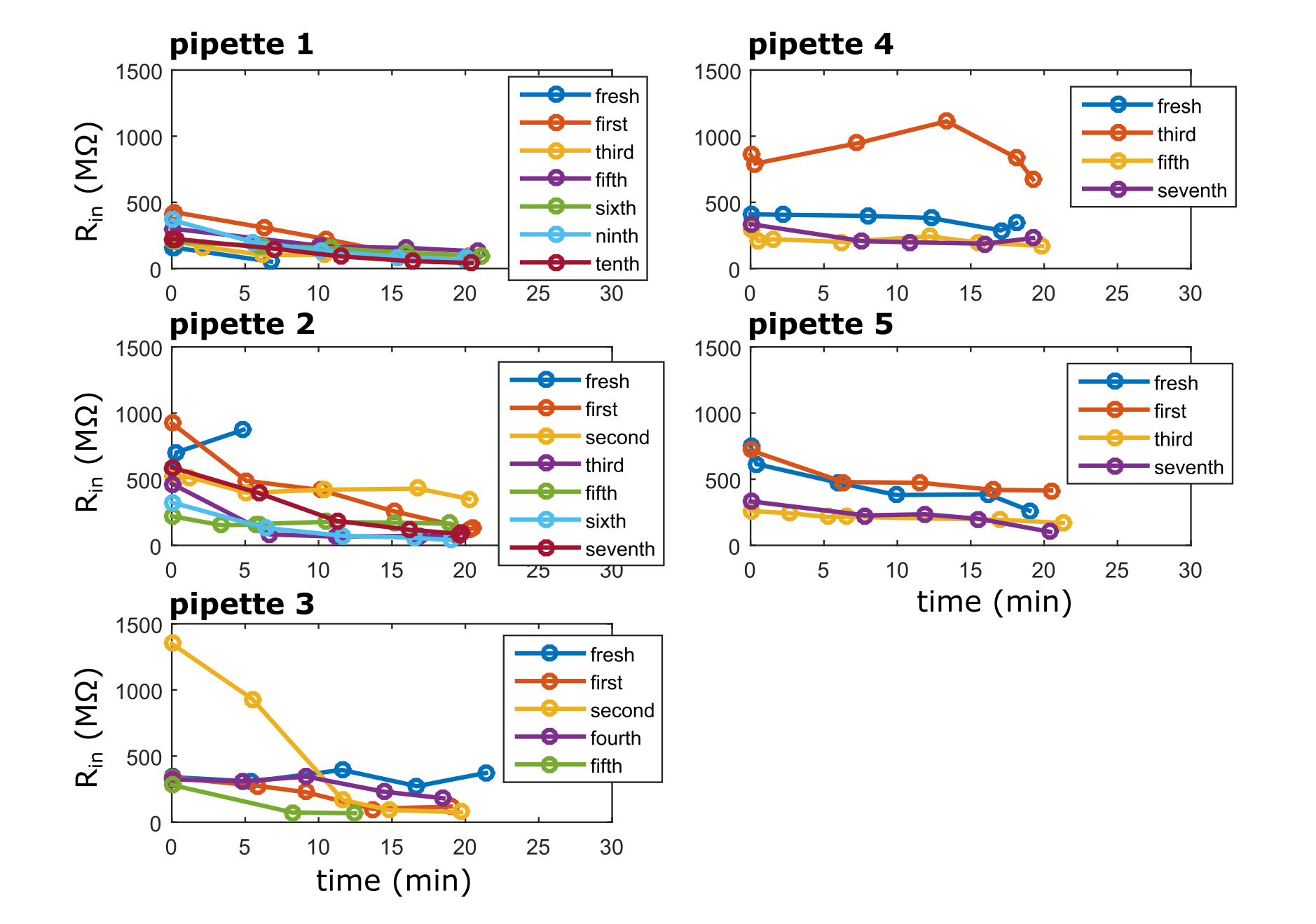


**SUPPLEMENTARY FIGURE 4**: Input resistance of neurons in brain slices. In most cases input resistance did not change dramatically over time for fresh pipettes as well as for pipettes reused 1-10 times, indicating stable whole-cell recordings.


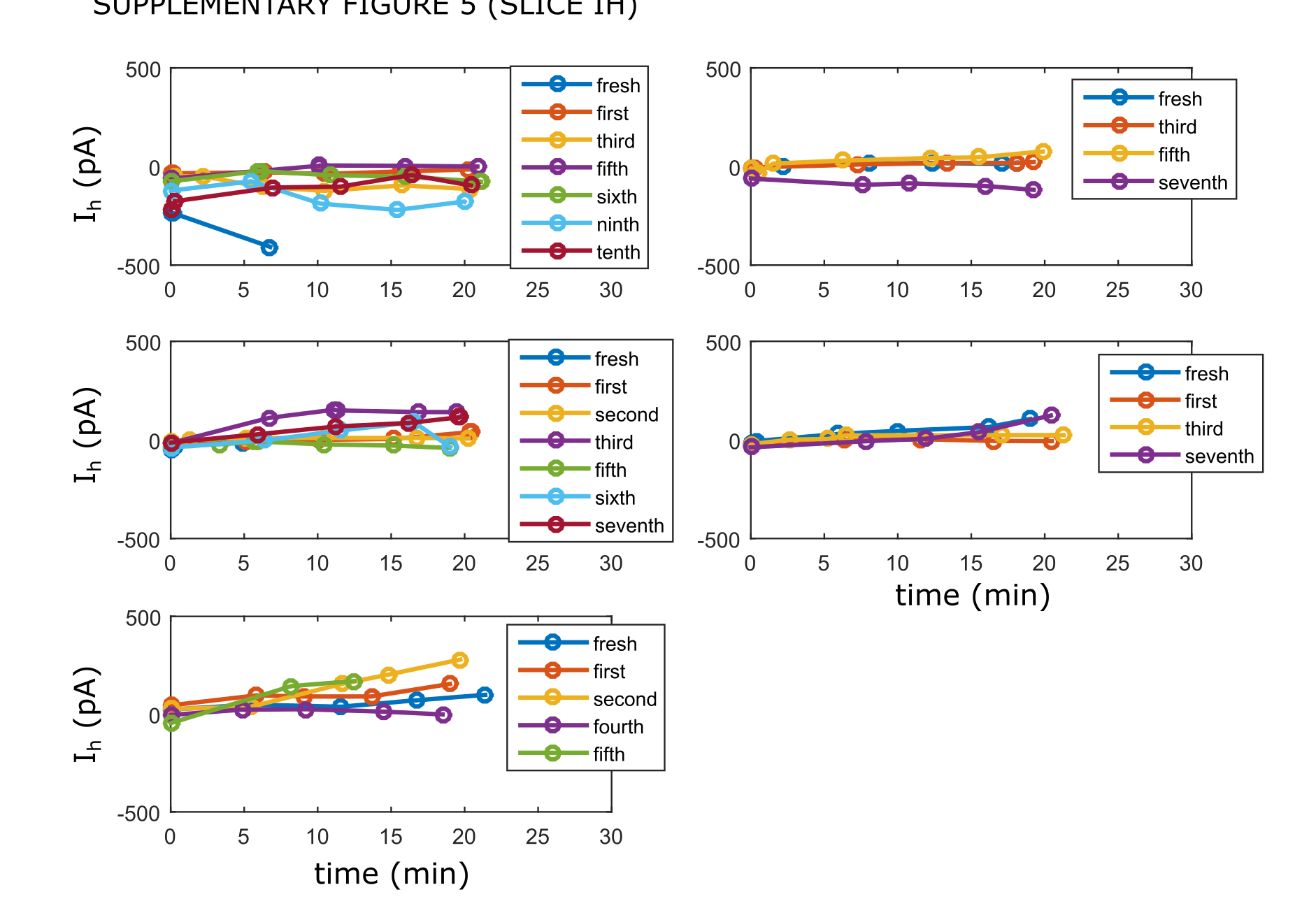


**SUPPLEMENTARY FIGURE 5**: Holding current of neurons in brain slices. In most cases the holding current did not change dramatically over time for fresh pipettes as well as for pipettes reused 1-10 times, indicating stable whole-cell recordings.


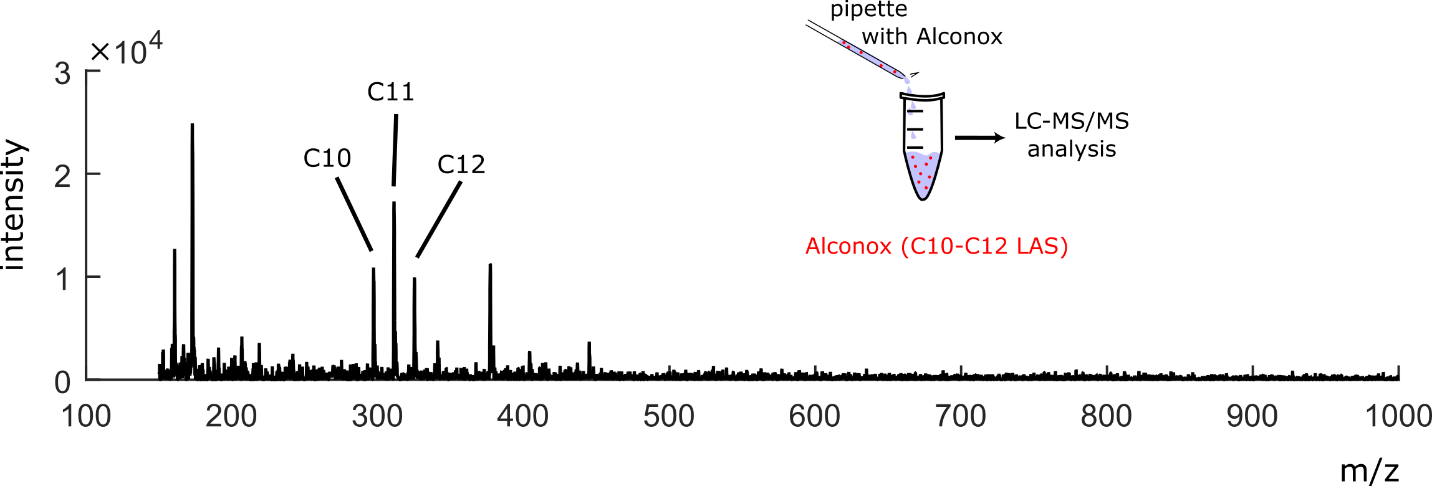


**SUPPLEMENTARY FIGURE 6**: ESI-MS spectrum characterization of pipettes containing 67 μg/ml Alconox dissolved in DI water. The main cytotoxic components of Alconox, C10-C12 LAS compounds were identified in the solution (C10: expected m/z: 297.1, found: 297.1; C11: expected m/z: 311.2, found: 311.1; C12: expected m/z: 325.2, found: 325.2).


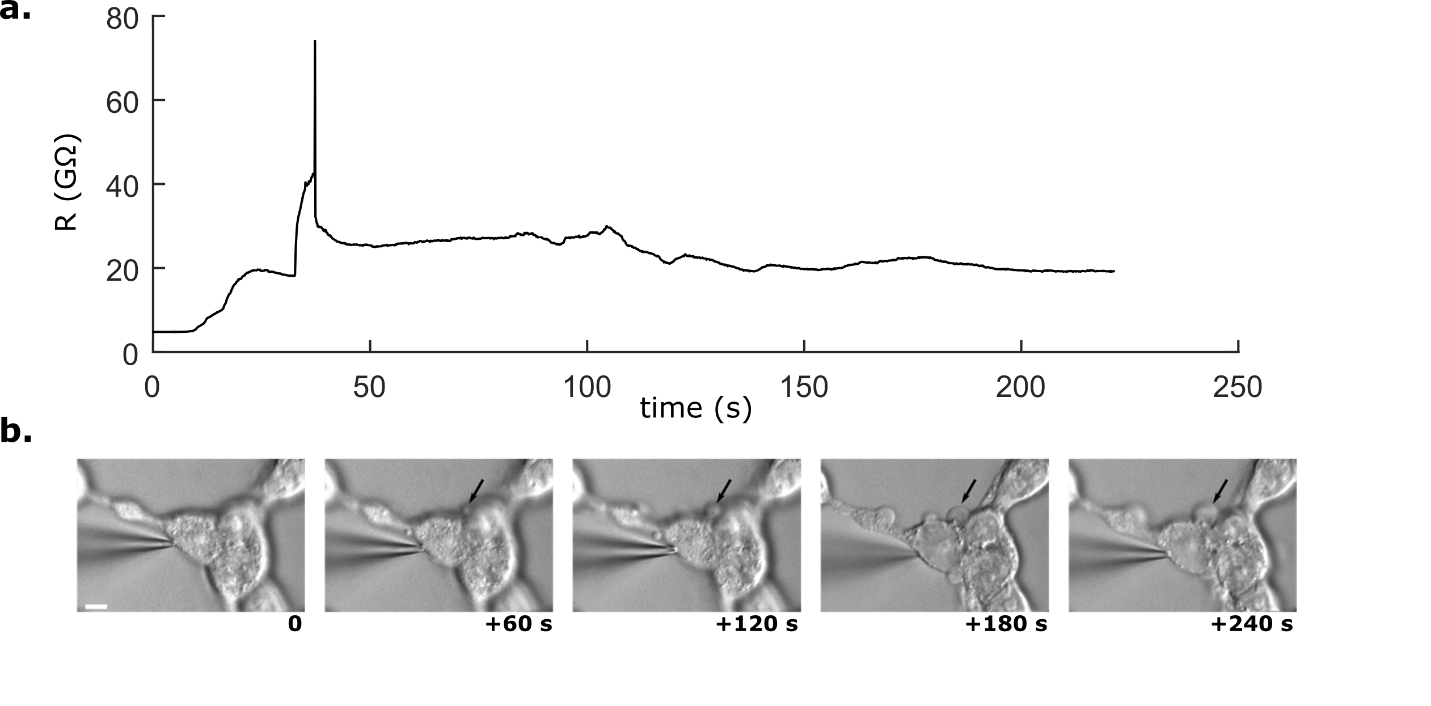


**SUPPLEMENTARY FIGURE 7**: A pipette containing a high dose (10 mg/mL) of Alconox damages the target cell during a patch attempt. **a**. A gigaseal fails to form after >3 min, suggesting that Alconox is destroying cell membrane. **b**. Cell apoptosis during the gigaseal process is evident. Arrow indicates blebs forming on the cell. Scale bar: 10 μm.

**SUPPLEMENTARY VIDEO 1**: Time-lapse video (~33 minutes) of unattended in-vitro patcherBot operation. Green light around microscope indicates successful whole-cell recording. A single pipette is used. Over the course of the video, the patcherBot obtains 9 gigaseals and 6 whole-cell recordings out of 10 attempts.

**SUPPLEMENTARY VIDEO 2**: Time-lapse video (~35 minutes) of unattended in-vivo patcherBot operation. Inset on bottom right shows the experimental preparation (craniotomy in mouse barrel cortex) and the wash and rinse baths. A single pipette is used. Over the course of the video, the patcherBot obtains 4 gigaseals and 3 whole-cells out of 5 attempts.

| **Cleaning solution** | **Source** | **Reference/Protocol** |
| --- | --- | --- |
| Bleach (8.25% Sodium hypochlorite) | The Clorox Company | (Kao et al. 2012) |
| Triton X-100 | X100-5mL (Sigma-Aldrich) | (Koley and Bard 2010) |
| Acetone | BDH1101 (VWR) | (Jamur and Oliver 2010) |
| Phosopolipase A_2_ (PLA, 0.0001% w/v in 10 mM HEPES buffer) | P7778 (Sigma-Aldrich) | (Beneš et al. 2004) |
| Sodium Dodecylbenzenesulfonate (SDS, 1% w/v) | TCD0990 (VWR) | (Kinoshita et al. 2009) |
| Alconox (2% w/v) | Alconox Inc. | Alconox User Manual – Alconox Inc. |

**SUPPLEMENTARY TABLE 1:** Pipette cleaning solutions

*References*

**Beneš M**, **Billy D**, **Benda A**, **Speijer H**, **Hof M**, **Hermens WT**. Surface-Dependent Transitions during Self-Assembly of Phospholipid Membranes on Mica, Silica, and Glass. *Langmuir* 20: 10129–10137, 2004.

**Jamur M**, **Oliver C**. Permeabilization of Cell Membranes [Online]. In: *Immunocytochemical Methods and Protocols*, edited by Oliver C, Jamur MC. Humana Press, p. 63–66. http://dx.doi.org/10.1007/978-1-59745-324-0_9 [14 May 2016].

**Kao L**, **Abuladze N**, **Shao XM**, **McKeegan K**, **Kurtz I**. A new technique for multiple re-use of planar patch clamp chips. *J Neurosci Methods* 208: 205–210, 2012.

**Kinoshita E**, **Kinoshita-Kikuta E**, **Koike T**. Separation and detection of large phosphoproteins using Phos-tag SDS-PAGE. *Nat Protoc* 4: 1513–1521, 2009.

**Koley D**, **Bard AJ**. Triton X-100 concentration effects on membrane permeability of a single HeLa cell by scanning electrochemical microscopy (SECM). *Proc Natl Acad Sci* 107: 16783–16787, 2010.
